# Spontaneous regression of ALK fusion protein-positive non-small cell lung carcinoma: a case report and review of the literature

**DOI:** 10.1186/s12890-020-01249-w

**Published:** 2020-08-06

**Authors:** Maria Walls, Gerard M. Walls, Jacqueline A. James, Kyle T. Crawford, Hossam Abdulkhalek, Tom B. Lynch, Aaron J. Peace, Terry E. McManus, O. Rhun Evans

**Affiliations:** 1 Centre for Medical Education, Queen’s University Belfast, Belfast, Northern Ireland; 2 Clinical Oncology Department, Cancer Centre Belfast City Hospital, Belfast Health & Social Care Trust, Belfast, Northern Ireland; 3 Patrick G Johnston Centre for Cancer Research, Queen’s University Belfast, Belfast, Northern Ireland; 4 Cellular Pathology Department, Belfast Health & Social Care Trust, Belfast, Northern Ireland; 5 Precision Medicine Centre of Excellence, Health Sciences Building, Queen’s University Belfast, Belfast, Northern Ireland; 6 Medical Oncology Department, North West Cancer Centre, Western Health & Social Care Trust, Derry, Northern Ireland; 7 Cardiology Department, Altnagelvin Hospital, Western Health & Social Care Trust, Derry, Northern Ireland; 8 Clinical Translational Research & Innovation Centre, Altnagelvin Hospital, Western Health & Social Care Trust, Derry, Northern Ireland; 9 Respiratory Department, South West Acute Hospital, Western Health & Social Care Trust, Enniskillen, Northern Ireland

**Keywords:** Non-small cell lung cancer, ALK rearrangement, Spontaneous regression, Radiotherapy, Embolism, Cancer immunity, Stroke, Electric therapy, DC cardioversion

## Abstract

**Background:**

ALK-rearrangement is observed in < 5% non-small cell lung cancer (NSCLC) cases and prior to the advent of oral tyrosine kinase inhibitors, the natural history of oncogenic NSCLC was typically poor. Literature relating to regression of treatment-naïve NSCLC is limited, and regression without treatment has not been noted in the ALK-rearranged sub-population.

**Case presentation:**

A 76 year old ‘never smoker’ female with an ALK-rearranged left upper lobe T2 N0 NSCLC experienced a stroke following elective DC cardioversion for new atrial fibrillation. Following a good recovery, updated imaging demonstrated complete regression of the left upper lobe lesion and a reduction of the previously documented mediastinal lymph node. Remaining atelectasis was non-avid on repeat PET-CT imaging, 8 months from the baseline PET-CT. When the patient developed new symptoms 6 months later a further PET-CT demonstrated FDG-avid local recurrence. She completed 55 Gy in 20 fractions but at 18 months post-radiotherapy there was radiological progression in the lungs with new pulmonary metastases and effusion and new bone metastases. Owing to poor performance status, she was not considered fit for targeted therapy and died 5 months later.

**Conclusion:**

All reported cases of spontaneous regression in lung cancer have been collated within. Documented precipitants of spontaneous regression across tumour types include biopsy and immune reconstitution; stroke has not been reported previously. The favourable response achieved with radical radiotherapy alone in this unusual case of indolent oncogenic NSCLC reinforces the applicability of radiotherapy in locally advanced ALK-rearranged tumours, in cases not behaving aggressively. As a common embolic event affecting the neurological and pulmonary vasculature is less likely, an immune-mediated mechanism may underpin the phenomenon described in this patient, implying that hitherto unharnessed principles of immuno-oncology may have relevance in oncogenic NSCLC. Alternatively, high electrical voltage applied percutaneously adjacent to the tumour during cardioversion in this patient may have induced local tumour cell lethality.

## Background

The clinical phenotype of non-small cell lung cancer (NSCLC) with the fusion gene echinoderm microtubule associated protein like 4 (EML4) - anaplastic lymphoma kinase (ALK), is characterised by early metastasis and poor prognosis in comparison to tumours without a known oncogenic driver [1]. ALK rearrangements are more common in younger, ‘never smoker’ and ‘light smoker’ patients [2] and multiple chromosomal rearrangements have been described [3]. ALK rearrangements are reportedly mutually exclusive with epidermal growth factor receptor (EGFR) and Kirsten rat sarcoma (KRAS) mutations [4].

Curative treatment options include surgery and radiotherapy, although inferior outcomes have been noted in comparison with cases where ALK rearrangement is not detected [5–7]. In advanced disease where radical interventions are not possible, targeted oral tyrosine kinase inhibitors offer improved outcomes over cytotoxic therapy [8]. Next generation targeted agents have improved efficacy and toxicity profiles [9] but clinical trials of immune checkpoint inhibitors have shown reduced efficacy in this small subpopulation [10].

Spontaneous regression (SR) of cancer, defined as at least partial disappearance of cancer without medical treatment, occurs in approximately 1 in 100,000 cases [11]. Most reported cases relate to melanoma, or haematological primaries, and are commonly attributed to the immune system [12]. Regression of untreated metastases following radiotherapy to the primary, the abscopal effect, is currently under investigation, with augmentation by systemic immunotherapy particularly in focus [13]. Regression of an oncogene-associated NSCLC without treatment has not been reported in the literature previously. The ALK-rearranged clinical case described herein according to the Case Report (CARE) guidance [14], underwent SR, sustained for at least 10 months, and radical radiotherapy subsequently on local relapse.

## Case presentation

**Fig. 1:**
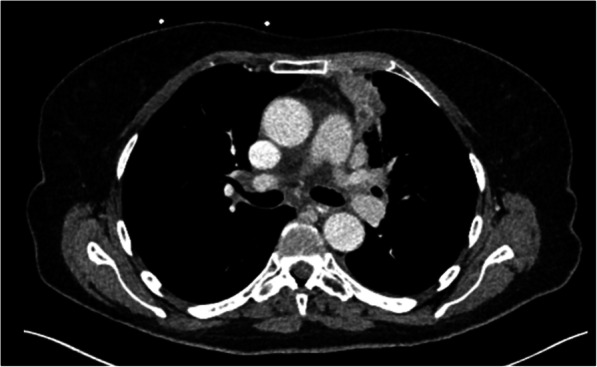
CT appearance of left upper lobe primary tumour

A 76 year old ‘never smoker’ female with no past medical history was diagnosed with locally advanced NSCLC during investigations for a community-acquired lower respiratory tract infection. The Medical Research Council (MRC) Dyspnoea Score was 3 and there was a dry cough. Computed tomography (CT) of the chest demonstrated a 4.5 cm (anterior-posterior) × 4.1 cm (craniocaudal) left lung upper lobe mass with abutment of the mediastinal pleura and distal atelectasis and pneumonitis (Fig. 1).

An 8 mm ipsilateral lymph node was visible at station 10. Multiple sub-centimetre lung nodules were noted throughout the right lung. Histological and immunohistochemical assessment of core biopsies from the primary lesion via bronchoscopy favoured the adenocarcinoma subtype of NSCLC (see Table 1). The molecular analysis revealed ALK fusion protein overexpression along with ALK rearrangement. This result is in keeping with an ALK rearranged adenocarcinoma. The main lesion had an SUV_max_ of 16 on ^18^fluorodeoxyglucose positron emission topography-CT (PET-CT) imaging and no other lesions were avid. Following a review of the imaging at the multidisciplinary meeting (MDM), staging was offered at T2 N0 M0 (TNM 8 [15]), and in light of the patient’s fitness, radical treatment was recommended.

**Table 1:** Pathological evaluation of core biopsy samples

Histological Analysis		Molecular Analysis	
**TTF-1**	Weak focal positivity	**EGFR PCR**	Insufficient DNA
**Nap A**	Weak focal positivity	**ALK fusion protein IHC**	Detected
**CK 7**	Positive	**ALK rearrangement ISH**	Detected
**CK 20**	Positive	**PD-L1 IHC**	Not detected
**CA 19–9**	Positive		
**CDX-2**	Negative		

(TTF-1 = thyroid transcription factor 1; Nap A = napsin A; CK 7 = cytokeratin-7; CK 20 = cytokeratin-20; CA 19.9 = carbohydrate antigen 19–9; CDX2 = caudal type homeobox 2; PCR = polymerase chain reaction; IHC = immunohistochemistry; ISH = in situ hybridisation; PD-L1 = programmed death ligand 1)

**Fig. 2:**
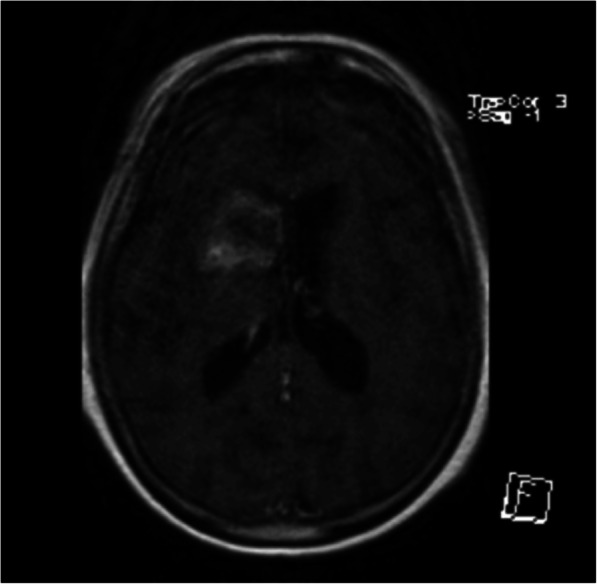
Axial T1-weighted MRI demonstrating right sided MCA territory infarction

During the assessment period for a primary lobectomy, the patient developed symptomatic atrial fibrillation. She underwent a successful direct current (DC) cardioversion and was discharged on edoxaban. Three weeks later the patient was noted to be in atrial fibrillation once more during an inpatient admission for the management of chest sepsis, for which she was discharged on digoxin. She was electively admitted to the Cardiology ward 6 weeks later for a second DC cardioversion procedure. Under conscious sedation, the patient received one synchronised shock of 120 J delivered via anterior-posterior paddles ie one placed at the left parasternal edge, one at the corresponding position on the patient’s back. One week post-procedure the patient attended the Emergency Department complaining of dysarthria and left-sided hemiparesis. CT and magnetic resonance imaging (MRI) of the brain confirmed the presence of a dense right-sided middle cerebral artery territory infarction (see Fig. 2). The patient was in sinus rhythm, transthoracic echocardiography was unremarkable and mild bilateral carotid atheroma only was noted on ultrasonography (< 50% stenosis), suggestive for a stroke secondary to a delayed cardiogenic embolus related to atrial fibrillation, despite anticoagulation.

Five days later, haemorrhagic transformation of the stoke was detected on MRI following clinical deterioration. A 3.5 cm intracerebral haematoma was identified within the right basal ganglia, causing effacement of the right lateral ventricle frontal horn, which was managed conservatively. The clinical condition stabilised and both her speech and weakness improved with rehabilitation from the department of Stroke Medicine.

As the patient’s ECOG PS recovered to 2 and her breathlessness resolved, work-up for radical treatment resumed, given her ongoing determination to gain control over the cancer. Updated cross-sectional imaging demonstrated complete regression of the left upper lobe lesion and a reduction of the previously documented mediastinal lymph node. Remaining atelectasis had a maximum standard uptake value (SUV_max_) 2.7 on repeat PET-CT imaging (8 months since first PET). A review of the patient’s medications was undertaken searching for possible effects on FDG uptake, which was negative. The merits and risks of radical radiotherapy versus active surveillance were explored with the patient who elected to proceed with the latter.

**Fig. 3:**
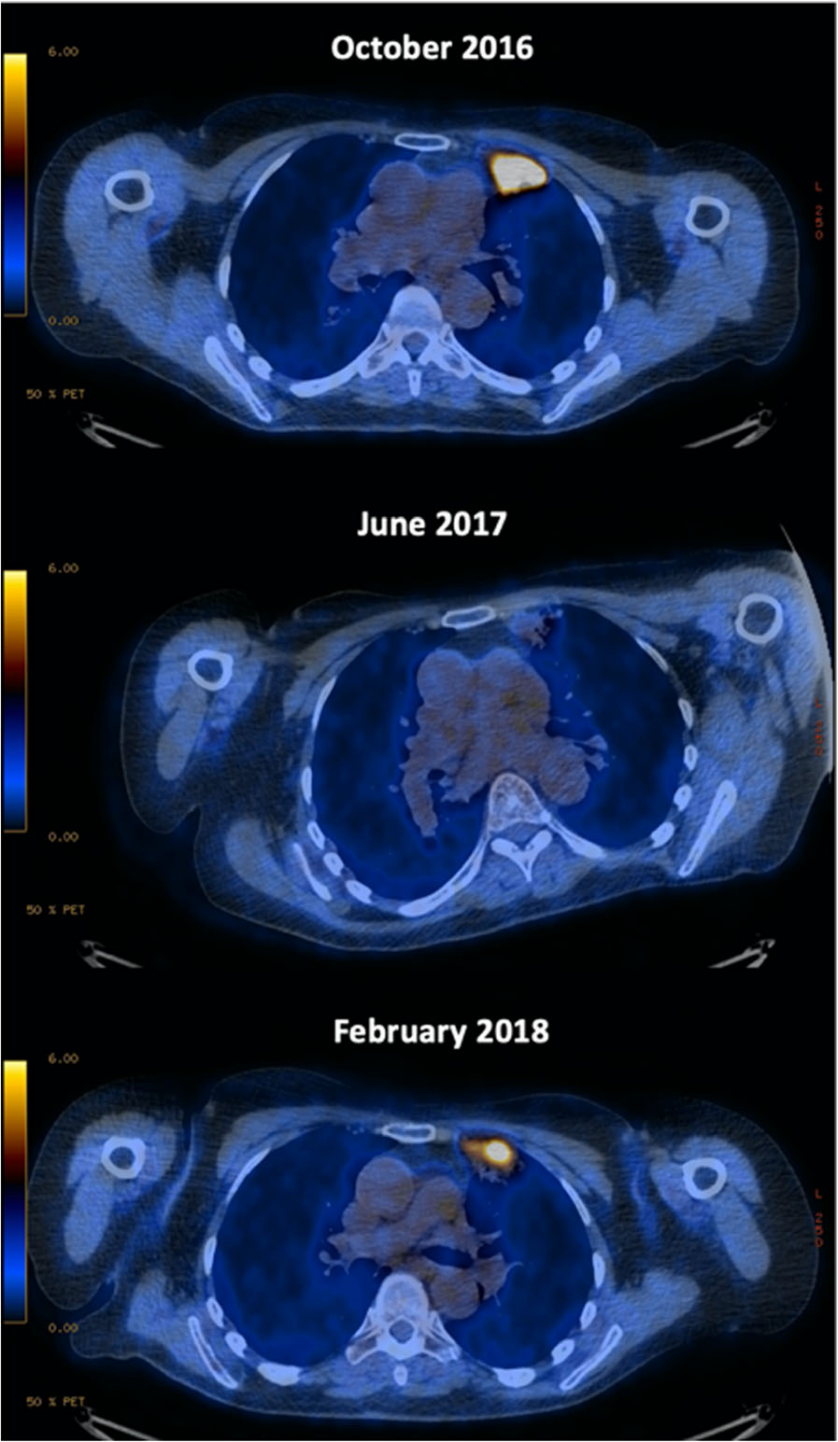
Serial PET-CT appearance of the left upper lobe lesion

Clinical review after 6 months of active surveillance, dry cough and mild dyspnoea were reported by the patient. Corresponding with the imaging findings, thoracic imaging with CT showed increased patchy parenchymal changes at the site of the previous left upper lobe lesion without associated hilar or mediastinal lymphadenopathy. Repeat PET-CT imaging demonstrated increased uptake (SUV_max_ 10.2) in a sub-pleural 4 cm mass in keeping with local relapse (see Fig. 3), and no additional sites of disease.

As the pulmonary function tests (FEV1 90% predicted; TLCO 80% predicted) were favourable and physical fitness had stabilised (ECOG PS 2) the patient was consented for a course of radical thoracic radiotherapy without chemotherapy. She completed 55 Gy in 20 fractions planned with the intensity modulated radiotherapy technique and delivered as 6 MV arc therapy, with daily online cone beam-CT image guidance, treating Monday to Friday for 4 weeks [16]. Target volumes were subject to peer review [17]. Tumour shrinkage was noted during routine offline imaging review.

There were no acute toxicities during routine clinical assessments on treatment, or at 6 weeks post-radiotherapy. On clinical review 4 months after treatment completion the patient was more frail and had continued respiratory symptoms. Around this time, the first radiological follow-up scan demonstrated radiation pneumonitis focally in left upper lobe. At 1 year the patient returned to ECOG PS 1 and the imaging demonstrated stable disease locally and no evidence of distant relapse. At 18 months post-radiotherapy there was radiologic progression in the lungs with new pulmonary nodules and effusion and new bone metastases correlating with new symptoms of dyspnoea, cough and back pain. Owing to poor performance status, she was not considered fit for systemic therapy including ALK-targeted therapy and was managed with multi-disciplinary best supportive care until her death 5 months later.

## Discussion and conclusions

SR is the partial or complete disappearance of a malignancy without medical treatment [12]. The incidence is estimated at 1 in 100,000 cases [11]. Kumar et al. [18] progressed the original definition recently, proposing that criteria below are met, and Ariza-Prota suggests that regression should be confirmed for at least 1 month [19].

- Partial or complete disappearance in the absence of systemic treatment or local treatment to local or distant disease
- No form of recent systemic therapy were administered
- The primary malignancy was made on histology, and metastatic lesions must have at least been confirmed on imaging

Most reported cases have involved primary tumours recognised for their immunogenicity. eg. melanoma, renal cell carcinoma and haematological cancers [20]. Evidence supporting immune (re-)activation as the mechanism for remission is accumulating, with both pre-clinical and clinical studies including cytokines and macrophages [21, 22]. Lung cancers undergo SR less frequently, possibly because they tend to be less immunogenic [23].

Two groups have previously collated pan-tumour SR cases – Everson and Boyd from 1900 to 1964, and on to 1987 by Challis. Limited clinical detail were available to the authors however, and oncology has been revolutionised by CT. Of the pooled lung cases (approximately twenty) it is likely most cases had small cell histology. All cases of SR in NSCLC published since 1987 are detailed in Table 2. Staging is recorded according to TNM 8 except in cases where insufficient information was available (marked with a *). Follow-up duration is recorded as the duration of time (months) from the first observation of regression to the end of follow-up, relapse at the regressed site of disease, or death (whichever came first). Triggers proposed specific to the case presented are listed, as are documented details of surveillance or treatment strategies. Cases of abscopal responses to RT [49], durable local control following R1 resections [50] and where treatment was given immediately prior to SR were excluded [51], and only articles in English were included.

**Table 2:** A summary of previous reports of SR in lung cancer

Authors	Age	Gender	Staging	Histology	Degree of Remission			Follow-Up Duration	Relapse or Death	Potential Trigger Identified	Previous / Subsequent Treatment / Outcomes Details
					Primary	Nodes	Metastases				
Jeong et al. 2019 [24]	64	M	T1c NX MX	Squamous cell carcinoma	Complete	Not applicable	Not applicable	16	Local relapse	Biopsy	• Neoadjuvant chemo on relapse • Lobectomy (ypT0 N2) • Adjuvant chemo + RT • Unknown follow-up
Matsui et al. 2018 [25]	56	F	T1a N2 M0	Squamous cell carcinoma	Partial	None	Not applicable	12	No	Biopsy	• Lobectomy (ypT1a N2) • No adjuvant treatment • Disease-free at 1 year
Ooi et al. 2018 [26]	77	M	T3 N1 M0	Not otherwise specified	Partial	Complete	Not applicable	24	No	None	• Surveillance ongoing
Ariza-Prota et al. 2018 [19]	82	M	T3 N3 M1c	Squamous cell carcinoma	Partial	Partial	Complete	12	No	Biopsy	• Palliative thoracic RT • Death 1 year later (MI)
Esplin et al. 2018 [27]	57	M	T1b N0 M0	Squamous cell carcinoma	Partial	Not applicable	Not applicable	Not reported	Not reported	Biopsy	Not reported
Miyoshi et al. 2017 [28]	80	M	Not reported	Adenocarcinoma	Partial	Not applicable	Not applicable	31	No	Biopsy	• Surveillance ongoing
Marques et al. 2017 [29]	75	M	T1b N0 M0	Adenocarcinoma	Complete	Not applicable	Not applicable	36	No	Biopsy	• Surveillance ongoing
Lopez-Pastorini et al. 2015 [30]	76	M	T3 N2 M0	Large cell carcinoma	Partial	Partial	Not applicable	84	No	Biopsy	• Surveillance ongoing
Choi et al. 2013 [31]	71	M	Not reported	Squamous cell carcinoma	Partial	Not applicable	Not applicable	Not reported	No	Tuberculosis	• Surveillance ongoing
Kappauf et al. 1997 [32]	61	M	TX NX M1b	Adenocarcinoma	Not Applicable	Not applicable	Complete	78	No	Biopsy	• Lobectomy 7/12 earlier
Park et al. 2016 [33]	79	M	TX NX M1a	Squamous cell carcinoma	Partial	Partial	Partial	14	No	Ginseng	Not reported
Ogawa et al. 2015 [34]	65	M	TX NX M1c	Not otherwise specified	Partial	Partial	Partial	Not reported	Not reported	Biopsy	• Palliative RT given for MSCC after SR had began
Kwint et al. 2015 [35]	80	M	T2a N3 M1b*	Not otherwise specified	Partial	Partial	Complete	6	No	None	Not reported
Cafferata et al. 2004 [36]	68	M	T1c N0 M0	Adenocarcinoma	Complete	Not applicable	Not applicable	48	No	None	Not reported
Chung et al. 2015 [37]	67	M	T4 N0 M1b	Squamous cell carcinoma	Partial	Not applicable	Not reported	13	No	Herbal medicine	Not reported
Menon et al. 2015 [38]	44	M	T1b N0 M1c	Not otherwise specified	Partial	Not applicable	Complete	60	No	HAART	• WBRT at diagnosis • HAART adherence increased
Hwang et al. 2013 [39]	62	M	T2a N3 M0	Not otherwise specified	Complete	Partial	Not applicable	14	No	None	• Declined all treatment
Mizuno et al. 2011 [40]	62	M	T1b N0 M0	Large cell carcinoma	Complete	Not applicable	Not applicable	6	Distant Relapse	Biopsy/Surgery	• Palliative chemo
Nakamura et al. 2009 [41]	71	M	T4 N0 M0	Adenocarcinoma	Disease Progression	Not applicable	Complete	34	No	Immunological	• Palliative RT to hilum
Pujol et al. 2007 [42]	75	F	Localised but no staging	Squamous cell carcinoma	Complete	Not applicable	Not applicable	18	No	Anti-Hu paraneoplastic syndrome	• Plasmapheresis
Miyazaki et al. 2007 [43]	74	M	TX N0 M1b	Adenocarcinoma	None	Not applicable	Complete	35	No	None	• Received radical RT to the primary eventually
Furukawa et al. 2011 [44]	56	M	T1 N0 M0*	Squamous cell carcinoma	Partial	Not applicable	Not applicable	2	No	Bullous disease	• Resected after 2 months
Gladwish et al. 2010 [45]	84	F	T3 N3 M0	Squamous cell carcinoma	Partial	Partial	Not applicable	12	No	None	• No treatment accepted
Yoon et al. 2019 [46]	74	F	T3 N1 M0	Not otherwise specified	Partial	Partial	Not applicable	9	No	Herbal medicine	• Progressed through 6 lines of palliative chemo • SR noted one year following cessation of chemo
Leo et al. 1999 [47]	59	M	T1c N1 M0	Large cell carcinoma	Complete	Not applicable	Not applicable	4	No	Inadequate vasculature	• Appearance of LN during SR • Lobectomy, bronchial sleeve resection and LN sampling performed
Tomizawa et al. 2014 [48]	85	F	T1c N0 M0	Large cell carcinoma	Partial	Not applicable	Not applicable	13	Local relapse	Immunological	• SR ended on commencing glucocorticoids for inflammatory arthritis • Lobectomy on relapse

A total of 25 cases were identified between 1987 and 2020. The mean age was 69 years. 4 cases were female, 32 males. Squamous cell carcinoma made up the greatest proportion (*n* = 10), followed by NSCLC not otherwise specified (*n* = 6) and adenocarcinomas (*n* = 6). All but three cases had SR observed in the primary mass. The SR was complete in 7 cases and partial in 15. The median duration of follow-up was 71 months (range 2–84). From cases where data was available, two cases experienced local relapse and one, distant relapse, and each of these cases had SR observed in the primary tumour. The most common proposed trigger was biopsy (*n* = 10), followed by immunological mechanisms (*n* = 5) and herbal medicine (*n* = 3).

The strengths of this clinical case report include serial PET imaging, complete follow-up and context of previous cases in exhaustive literature review. The weaknesses of this case report include the lack of repeat biopsy on the initial relapse following SR, and the lack of previous oncogenic NSCLC cases available for comparison.

ALK rearrangement is uncommon in NSCLC amongst the Western population, with a prevalence of approximately 5% [52]. This oncogene is associated with unregulated tyrosine kinase activity and neoplastic transformation of pulmonary epithelial tissue, with the development of signet ring cell morphology [53]. The diagnosis of ALK rearrangement is therefore detrimental for prognostication in NSCLC and effective treatment options are limited [52]. Given the ‘oncogene addiction’ responsible for the poor prognosis of ALK rearranged NSCLC, the case presented represents unusual biology. Unsurprisingly, SR has not been reported previously in the ALK rearranged subpopulation.

**Fig. 4:**
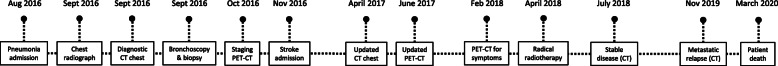
Timeline of clinical events

Local recurrence-free survival rate at 2 years following radical radiotherapy alone is offered at approximately 29% with modern treatment in the unselected NSCLC population [54], but this value is thought to be lower in oncogenic NSCLC [5, 7]. Local control was achieved for 24 months in the case outlined despite ALK rearrangement (see Fig. 4), also in keeping with atypical biology. There is a paucity of data on how radiotherapy should be optimised for oncogenic NSCLC. The role of ablative approaches in oncogenic NSCLC is the subject of the ongoing HALT trial in the UK [55].

Possible precipitants of SR have been purported by several investigators, of which the more common examples can be found in Table 3. No reports of stroke as a trigger for SR were identified in the NSCLC literature (see Table 2) and to the authors’ knowledge, no cases of SR following stroke have been documented in other primary tumours to date. One potential explanation for the SR observed in this case would be that an embolic shower affected the both the right carotid artery and the vasculature of the tumour, causing both regions to infarct. This is unlikely given that no patent foramen ovale was noted on echocardiogram. Separately, as stroke has been recognised as a highly stressful and inflammatory event [63], it is plausible that that molecular mimicry between the biological constituents of infarcted and/or haemorrhagic neural tissue and the oncogenic NSCLC microenvironment may have played a role in relegation of tumour volume.

**Table 3:** Purported triggers of SR

Mechanism	Potential Explanations
Biopsy	• Damage to supplying vasculature [29] • Immune response triggered by local inflammation [29]
Immunological	• NK activation [56] • Infection-related immune upregulation [57] • CD8^+^ cell infiltration [58]
Hormonal	• Down-regulation of tumour proliferation pathways [59]
Intrinsic regression	• Upregulation of apoptotic pathways [60] • Return of tumour cell differentiation [61] • Removal of carcinogen [62]

Finally, electrical pulses are known to reversibly increase the permeability of cells (known as electroporation) in vitro [64]. In oncology this has been used to improve the delivery of systemic anticancer therapy locally within tumours in vivo [65] and clinically [66], and as a stand alone treatment where even higher voltages induce apoptosis by irreversible cell membrane damage, known as irreversible electroporation (IRE) [67]. IRE has been shown to be safe for endobronchial tumours in animal studies [68] and small clinical studies have been undertaken [69]. The mechanism of the differential survival of normal tissues compared with tumour cells may be related to repair kinetics [70] or to modification of the immune system [71]. The high electrical voltage applied percutaneously adjacent to the left upper lobe tumour during the elective DC cardioversion of this patient for atrial fibrillation may have induced cellular lethality. To our knowledge there are no previous reports of (complete) regression of a primary lung tumour following DC cardioversion.

Given that treatment with targeted therapies can gain control of NSCLC by instigation of immunogenic cellular lethality [72], this case raises the possibility of undiscovered immune targets relevant for the treatment of oncogenic NSCLC. Further research is thereby warranted to elicit the full extent of the role of the immune system in oncogenic-driven tumours.

In conclusion, this is the first report of SR of NSCLC with a driver oncogene. The underlying biological mechanism is unclear, but temporally was related to electrical cardioversion and a subsequent embolic event. If SR was immune-mediated in this case, one hypothesis would be that hitherto unactionable immune checkpoints may be viable therapeutic targets in oncogenic lung cancers. Alternatively, further translational research into electrical therapy for lung cancer may be warranted. Concerted international academic effort will be required to collect cases of SR in NSCLC in order to unravel the underpinning biology.

## Data Availability

Not applicable.

## Abbreviations

ALK: Anaplastic lymphoma kinase
CT: Computed tomography
ECOG: Eastern Cooperative Oncology Group
EGFR: Epidermal growth factor receptor
EML4: Echinoderm microtubule associated protein like 4
FEV1: Forced expiratory volume in one second
KRAS: Kirsten rat sarcoma
MDM: Multidisciplinary meeting
MRC: Medical Research Council
MRI: Magnetic resonance imaging
NSCLC: Non-small cell lung cancer
PET: Positron emission topography-computed tomography
SR: Spontaneous regression
SUV_max_: Maximum standard uptake value
TLCO: Transfer factor for carbon monoxide

## References

[1] Yang P, Kulig K, Boland JM, Erickson-Johnson MR, Oliveira AM, Wampfler J (2012). Worse disease-free survival in never-smokers with <em>ALK</em>+ lung adenocarcinoma. J Thorac Oncol.

[2] Camidge DR, Kono SA, Flacco A, Tan AC, Doebele RC, Zhou Q (2010). Optimizing the detection of lung cancer patients harboring anaplastic lymphoma kinase (ALK) gene rearrangements potentially suitable for ALK inhibitor treatment. Clin Cancer Res.

[3] Rosas G, Ruiz R, Araujo JM, Pinto JA, Mas L (2019). ALK rearrangements: biology, detection and opportunities of therapy in non-small cell lung cancer. Crit Rev Oncol Hematol.

[4] Gainor JF, Varghese AM, Ou SHI, Kabraji S, Awad MM, Katayama R (2013). ALK rearrangements are mutually exclusive with mutations in EGFR or KRAS: an analysis of 1,683 patients with non-small cell lung cancer. Clin Cancer Res.

[5] Hayashi H, Okamoto I, Kimura H, Sakai K (2012). Clinical Outcomes of Thoracic Radiotherapy for Locally Advanced NSCLC with EGFR Mutations or EML4-ALK Rearrangement.

[6] Chaft JE, Dagogo-Jack I, Santini FC, Eng J, Yeap BY, Izar B (2018). Clinical outcomes of patients with resected, early-stage ALK-positive lung cancer. Lung Cancer.

[7] Boros A, Lacroix L, Lacas B, Adam J, Pignon JP, Caramella C (2017). Prognostic value of tumor mutations in radically treated locally advanced non-small cell lung cancer patients. Oncotarget..

[8] Solomon BJ, Mok T, Kim D-W, Wu Y-L, Nakagawa K, Mekhail T (2014). First-line Crizotinib versus chemotherapy in ALK-positive lung Cancer. N Engl J Med.

[9] Camidge DR, Kim HR, Ahn M-J, Yang JC-H, Han J-Y, Lee J-S (2018). Brigatinib versus Crizotinib in ALK-positive non–small-cell lung Cancer. N Engl J Med.

[10] Gainor JF, Shaw AT, Sequist LV, Fu X, Azzoli CG, Piotrowska Z (2016). EGFR mutations and ALK rearrangements are associated with low response rates to PD-1 pathway blockade in non-small cell lung cancer: a retrospective analysis. Clin Cancer Res.

[11] Cole WH (1981). Efforts to explain spontaneous regression of cancer. J Surg Oncol.

[12] Everson TC, Cole WH (1959). Spontaneous regression of malignant disease: guest editorial. J Am Med Assoc.

[13] Theelen WS, de Jong MC, Baas P (2020). Synergizing systemic responses by combining immunotherapy with radiotherapy in metastatic non-small cell lung cancer: the potential of the abscopal effect. Lung Cancer.

[14] Gagnier JJ, Kienle G, Altman DG, Moher D, Sox H, Riley D (2013). The CARE guidelines: consensus-based clinical case reporting guideline development. BMJ Case Rep.

[15] Detterbeck FC (2018). The eighth edition TNM stage classification for lung cancer: what does it mean on main street?. J Thorac Cardiovasc Surg.

[16] Walls GM, Lyons C, Jellett LJ, Evans R, Bedair A, Brady D (2019). Radiation Oncology: A Clinical Update from The North West Cancer Centre. Ulster Med J.

[17] Rooney KP, McAleese J, Crockett C, Harney J, Eakin RL, Young VAL (2015). The impact of colleague peer review on the radiotherapy treatment planning process in the radical treatment of lung cancer. Clin Oncol (R Coll Radiol).

[18] Kumar T, Patel N, Talwar A (2010). Spontaneous regression of thoracic malignancies. Respir Med.

[19] Ariza-Prota M, Martínez C, Casan P (2018). Spontaneous regression of metastatic squamous cell lung cancer. Clin Case Reports.

[20] Ricci SB, Cerchiari U (2010). Spontaneous regression of malignant tumors: importance of the immune system and other factors (review). Oncol Lett.

[21] Li A-J, Wu M-C, Cong W-M, Shen F, Yi B (2003). Spontaneous complete necrosis of hepatocellular carcinoma: a case report. Hepatobiliary Pancreat Dis Int.

[22] Ludovic Croxford J, Tang MLF, Pan MF, Huang CW, Kamran N, Phua CML (2013). ATM-dependent spontaneous regression of early Em-myc-induced murine B-cell leukemia depends on natural killer and T cells. Blood..

[23] Ferro S, Huber V, Rivoltini L (2018). Mechanisms of tumor immunotherapy , with a focus on thoracic cancers.

[24] Jeong CP, Yi J, Jeong SS, Lee KN (2019). Lymph Node Metastasis after Spontaneous Regression of Non-Small Cell Lung Cancer. Korean J Thorac Cardiovasc Surg.

[25] Matsui T, Mizuno T, Kuroda H, Sakakura N, Arimura T, Yatabe Y (2018). Spontaneous regression of lung squamous cell carcinoma with synchronous mediastinal progression: a case report. Thorac Cancer.

[26] Ooi KH, Cheo T, Soon GST, Leong CN (2018). Spontaneous regression of locally advanced nonsmall cell lung cancer: a case report. Med (United States).

[27] Esplin N, Fergiani K, Legare T, Stelzer J, Bhatti H, Ali S (2018). Spontaneous regression of small cell lung Cancer: a case report. J Clin Case Reports.

[28] Miyoshi Y, Takayashiki N, Satoh H (2017). Spontaneous regression of FDG/PET positive lung adenocarcinoma in an elderly man. Adv Respir Med.

[29] Marques C, Queiroga H, Marques M, Moura C (2017). Spontaneous regression of a pulmonary adenocarcinoma after core needle biopsy. Autops Case Reports.

[30] Lopez-Pastorini A, Plönes T, Brockmann M, Ludwig C, Beckers F, Stoelben E (2015). Spontaneous regression of non-small cell lung cancer after biopsy of a mediastinal lymph node metastasis: a case report. J Med Case Rep.

[31] Choi SM, Go H, Chung DH, Yim JJ (2013). Spontaneous regression of squamous cell lung cancer. Am J Respir Crit Care Med.

[32] Grandgirard J, Poinsot D, Krespi L, Nénon JP, Cortesero AM (2002). Costs of secondary parasitism in the facultative hyperparasitoid Pachycrepoideus dubius: does host size matter?. Entomol Exp Appl.

[33] Park YH, Park BM, Park SY, Choi JW, Kim SY, Kim JO (2016). Spontaneous regression in advanced squamous cell lung carcinoma. J Thorac Dis.

[34] Ogawa R, Watanabe H, Yazaki K, Fujita K, Tsunoda Y, Nakazawa K (2015). Lung cancer with spontaneous regression of primary and metastatic sites: a case report. Oncol Lett.

[35] van MK M, Dominic Snijders den H, Jose Belderbos KM (2015). Spontaneous regression of large cell carcinoma of the lung, a case report. Omi J Radiol.

[36] Cafferata MA, Chiaramondia M, Monetti F, Ardizzoni A (2004). Complete spontaneous remission of non-small-cell lung cancer: a case report. Lung Cancer.

[37] Chung C, Il PD, Kim SY, Kim JO, Jung SS, Park HS (2015). spontaneous regression of non-small cell lung cancer that progressed after multiple chemotherapies: a case report. Thorac Cancer.

[38] Menon MP, Eaton KD (2015). Spontaneous regression of non-small-cell lung cancer in AIDS after immune reconstitution. J Thorac Oncol.

[39] Hwang ED, Kim YJ, Leem AY, Ji AY, Choi Y, Jung JY (2013). Spontaneous regression of non-small cell lung cancer in a patient with idiopathic pulmonary fibrosis: a case report. Tuberc Respir Dis (Seoul).

[40] Mizuno T, Usami N, Okasaka T, Kawaguchi K, Okagawa T, Yokoi K (2011). Complete spontaneous regression of non-small cell lung cancer followed by adrenal relapse. Chest..

[41] Nakamura Y, Noguchi Y, Satoh E, Uenaka A, Sato S, Kitazaki T (2009). Spontaneous remission of a non-small cell lung cancer possibly caused by anti-NY-ESO-1 immunity. Lung Cancer.

[42] Pujol JL, Godard AL, Jacot W, Labauge P (2007). Spontaneous complete remission of a non-small cell lung cancer associated with anti-Hu antibody syndrome. J Thorac Oncol.

[43] Miyazaki K, Masuko H, Satoh H, Ohtsuka M (2007). Lung cancer with spontaneous regression of scalp metastasis. Respir Med Extra.

[44] Furukawa M, Oto T, Yamane M, Toyooka S, Kiura K, Miyoshi S (2011). Spontaneous regression of primary lung cancer arising from an emphysematous bulla. Ann Thorac Cardiovasc Surg.

[45] Gladwish A, Clarke K, Bezjak A (2010). Spontaneous regression in advanced non-small cell lung cancer. BMJ Case Rep.

[46] Yoon HY, Park HS, Cho MS, Shim SS, Kim Y, Lee JH (2019). Spontaneous remission of advanced progressive poorly differentiated non-small cell lung cancer: a case report and review of literature. BMC Pulm Med.

[47] Leo F, Nicholson AG, Hansell DM, Corrin B, Pastorino U (1999). Spontaneous regression of large-cell carcinoma of the lung--a rare observation in clinical practice. Thorac Cardiovasc Surg.

[48] Tomizawa K, Suda K, Takemoto T, Iwasaki T, Sakaguchi M (2015). Progression after spontaneous regression in lung large cell neuroendocrine carcinoma : Report of a curative resection Case report. Thorac Cancer.

[49] Siva S, Callahan J, MacManus MP, Martin O, Hicks RJ, Ball DL (2013). Abscopal [corrected] effects after conventional and stereotactic lung irradiation of non-small-cell lung cancer. J Thorac Oncol.

[50] Smith RA (1971). Cure of lung cancer from incomplete surgical resection. Br Med J.

[51] Bell JW (1970). Possible immune factors in spontaneous regression of bronchogenic carcinoma. Ten year survival in a patient treated with minimal (1,200 r) radiation alone. Am J Surg.

[52] Chia PL, Dobrovic A, Dobrovic A, John T (2014). Prevalence and natural history of ALK positive non-small-cell lung cancer and the clinical impact of targeted therapy with ALK inhibitors. Clin Epidemiol.

[53] Sasaki T, Rodig SJ, Chirieac LR, Ja PA (2010). Current perspective The biology and treatment of EML4-ALK non-small cell lung cancer.

[54] Joo JH, Song SY, Kim SS, Jeong Y, Jeong SY, Choi W (2015). Definitive radiotherapy alone over 60 Gy for patients unfit for combined treatment to stage II-III non-small cell lung cancer: retrospective analysis. Radiat Oncol.

[55] McDonald F, Hanna GG (2018). Oligoprogressive oncogene-addicted lung Tumours: does stereotactic body radiotherapy have a role? Introducing the HALT Trial. Clin Oncol.

[56] Kawamura T, Seki S, Takeda K, Narita J, Ebe Y, Naito M (1999). Protective effect of NK1.1(+) T cells as well as NK cells against intraperitoneal tumors in mice. Cell Immunol.

[57] Nauts HC (1989). Bacteria and cancer--antagonisms and benefits. Cancer Surv.

[58] Haruki T, Nakamura H, Taniguchi Y, Miwa K, Adachi Y, Fujioka S (2010). Spontaneous regression of lung adenocarcinoma: report of a case. Surg Today.

[59] Hercbergs A, Leith J (1993). Spontaneous remission of metastatic lung Cancer following myxedema coma—an apoptosis- related phenomenon?. J Natl Cancer Inst.

[60] Hoehner JC, Hedborg F, Wiklund HJ, Olsen L, Pahlman S (1995). Cellular death in neuroblastoma: in situ correlation of apoptosis and bcl-2 expression. Int J Cancer.

[61] Stoll BA (1992). Spontaneous regression of cancer: new insights. Biotherapy..

[62] Montalban C, Santon A, Boixeda D, Bellas C (2001). Regression of gastric high grade mucosa associated lymphoid tissue (MALT) lymphoma after helicobacter pylori eradication. Gut..

[63] Jin R, Yang G, Li G (2010). Inflammatory mechanisms in ischemic stroke : role of inflammatory cells. J Leukoc Biol.

[64] Jiang C, Davalos RV, Bischof JC (2015). A review of basic to clinical studies of irreversible electroporation therapy. IEEE Trans Biomed Eng.

[65] Bhutiani N, Agle S, Li Y, Li S, Martin RCG (2016). Irreversible electroporation enhances delivery of gemcitabine to pancreatic adenocarcinoma. J Surg Oncol.

[66] Matthiessen LW, Chalmers RL, Sainsbury DCG, Veeramani S, Kessell G, Humphreys AC (2011). Management of cutaneous metastases using electrochemotherapy. Acta Oncol (Madr).

[67] Wagstaff PGK, Buijs M, van den Bos W, de Bruin DM, Zondervan PJ, de la Rosette JJMCH (2016). Irreversible electroporation: state of the art. Onco Targets Ther.

[68] Kodama H, Vroomen LG, Ueshima E, Reilly J, Brandt W, Paluch LR (2018). Catheter-based endobronchial electroporation is feasible for the focal treatment of peribronchial tumors. J Thorac Cardiovasc Surg.

[69] Thomson KR, Cheung W, Ellis SJ, Federman D, Kavnoudias H, Loader-Oliver D (2011). Investigation of the safety of irreversible electroporation in humans. J Vasc Interv Radiol.

[70] Jourabchi N, Beroukhim K, Tafti BA, Kee ST, Lee EW (2014). Irreversible electroporation (NanoKnife) in cancer treatment. Gastrointest Interv.

[71] Sugimoto K, Kakimi K, Takeuchi H, Fujieda N, Saito K, Sato E (2019). Irreversible Electroporation versus Radiofrequency Ablation: Comparison of Systemic Immune Responses in Patients with Hepatocellular Carcinoma. J Vasc Interv Radiol.

[72] Liu P, Zhao L, Pol J, Levesque S, Petrazzuolo A, Christina P (2019). Crizotinib-induced immunogenic cell death in non-small cell lung cancer.

